# Within-Crop Air Temperature and Humidity Outcomes on Spatio-Temporal Distribution of the Key Rose Pest *Frankliniella occidentalis*

**DOI:** 10.1371/journal.pone.0126655

**Published:** 2015-05-26

**Authors:** Hicham Fatnassi, Jeannine Pizzol, Rachid Senoussi, Antonio Biondi, Nicolas Desneux, Christine Poncet, Thierry Boulard

**Affiliations:** 1 INRA, UMR1355, ISA, 400 Route des chappes, 06903, Sophia Antipolis, France; 2 INRA, UR 0546, Biostatistique et Processus Spatiaux, F-84914, Montfavet, France; 3 University of Catania, Di3A, Via Santa Sofia 100, 95123, Catania, Italy; Ghent University, BELGIUM

## Abstract

*Frankliniella occidentalis* (Pergande) is a key pest of various crops worldwide. In this study, we analyse the dependence of the infestation of this pest on spatially distributed micro climatic factors in a rose greenhouse. Despite the importance of this subject, the few existing studies have been realized in laboratory rather than in greenhouse conditions. However, recent progress on greenhouse microclimate characterisation has highlighted the strong indoor climate heterogeneity that may influence the within-crop pest distribution. In this study, both microclimate (air temperature and humidity) and thrips distribution were simultaneously mapped in a rose greenhouse. The measurements were sensed in a horizontal plane situated at mid-height of the rose crop inside the greenhouse. Simultaneously, thrips population dynamics were assessed after an artificial and homogeneous infestation of the rose crop. The spatio-temporal distribution of climate and thrips within the greenhouse were compared, and links between thrips infestation and climatic conditions were investigated. A statistical model was used to define the favourable climate conditions for thrips adults and larvae. Our results showed that (i) the air temperature and air humidity were very heterogeneously distributed within the crop, (ii) pest populations aggregated in the most favourable climatic areas and (iii) the highest population density of thrips adults and larvae were recorded at 27°C and 22°C for temperature and 63% and 86% for humidity, respectively. These findings confirm, in real rose cropping conditions, previous laboratory studies on the *F*. *occidentalis* climatic optimum and provide a solid scientific support for climatic-based control methods against this pest.

## Introduction

Thrips (Thysanoptera: Thripidae) are important opportunistic pests of various cultivated crops around the world [1,2,3]. More specifically, *Frankliniella occidentalis* (Pergande) is a highly polyphagous phytophagous insect and it is considered among the most important pests of greenhouse vegetable and ornamental crops worldwide [4,5,6]. Native to North America [7], it is an invasive species in Europe. It was accidentally introduced into Europe in the early 80s, and reached France in 1986 [4–8]. It causes considerable damage to commercial flower crops, through direct feeding on marketable plant organs, i.e., flowers or flower buds, and by plant-viruses vectoring [3,9,10]. Damage related to *F*. *occidentalis* infestation varies according to the host plants. On rose crops such as necroses and deformations have been observed on young leaves, but it is mainly on flowers that effects are the most serious [11–12].

The most common practices used for controlling thrips pests rely on the chemical approaches [1,3,14]. Unfortunately, the intensive horticulture cropping activity selected many *F*. *occidentalis* insecticide-resistant populations [14,15,16]. Moreover, the widespread insecticide use is often associated with multiple human health [17] and environmental issues [18, 19, 20]. This exhorts for developing environmentally sound and sustainable management pest strategies restraining the use of insecticides [6–21]. For this purpose, a thorough knowledge on pest biology and ecology in its environment is strongly needed [22,23,24]. In particular, air temperature and humidity conditions closely surrounding the thrips habitats are known as important factors influencing their populational biology.

Several laboratory studies have shown that various *F*. *occidentalis* life history traits, such as development duration, longevity, sex-ratio and fertility, can largely depend on the climatic conditions tried out in laboratory [25,26,27]. Thereby, Robb and Parella [28] showed the longevity and fertility dependence of *F*. *occidentalis* on air temperature, finding that the highest longevity occurs at 20°C whereas the highest fertility is achieved between 20 and 27.2°C. However, all these life-table studies were carried out in strictly controlled laboratory conditions, and not in the actual cropping settings, in which the climate is often very heterogeneous [29]. This heterogeneity has been recently demonstrated in several studies evaluating the greenhouse climate variations using Computational Fluid Dynamics (CFD) modelling [30,31,32,33]. This modeling approach details the airflows and heat exchanges by numerically solving the equations governing the air dynamic in the greenhouse.

In this context, the deepening of our knowledge concerning the relation between fluctuating micro-climate cropping conditions and thrips population dynamic responses is crucial in any attempt of pest management. So, our study aimed at characterizing/quantifying the effect of air temperature and humidity on the spatio-temporal distribution of *F*. *occidentalis* populations in a protected rose cropping system.

## Materials and Methods

### Experimental greenhouse and climatic measurements

The experiment was performed during the summer of 2008 at the facilities of the INRA research center in Sophia Antipolis, South-Eastern France (43° 36' 44.9'' N latitude, 07° 04' 40.4'' E longitude and 125 m altitude). We used a compartment of about 40 m² (6.2 m wide and 6.2 m long) in a rose experimental glasshouse. The compartment was naturally ventilated by roof opening oriented east-west and equipped with an insect-proof screen.

**Fig 1:**
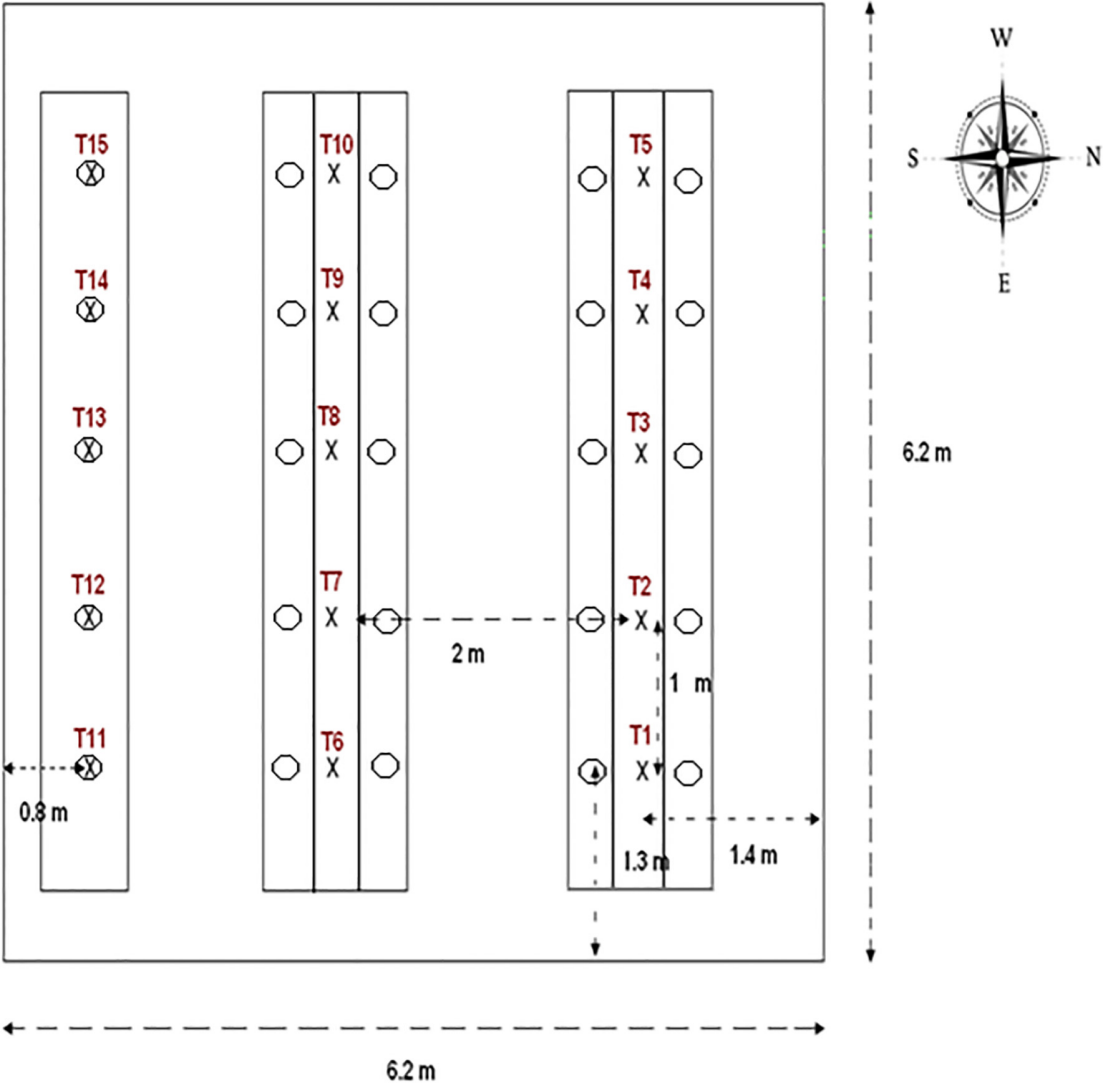
Layout of temperature and humidity sensors (x) and sampled plants (o) locations inside the experimental greenhouse.

15 individual air temperature and humidity measurements were set up at equal distance throughout the greenhouse area in a horizontal plane at the level of the rose buds (1.3m height) (Fig 1) [30,31]. The thermo-hygrometer sensors (model EE06, Intertechnique, Plaisir en Yvelines, France) were connected to two data loggers (model CR 23, Campbell, Shepshed, United Kingdom) on which data were transferred every second. The average values were recorded every 15 min before being downloaded and processed in a computer for displaying air temperature and humidity contours plots using 3DField software.

### Biological materials and samplings

The plants used in the experiment were young pest- and pesticide-free rose plants, *Rosa hybrida* L. of the thrips-susceptible variety Aubade (*Nirp International*), obtained using in vitro synthesis cultivation. Plants were grown on new rock wool slabs in single rows. Five rows of 25 rose plants (25±2 cm high) were transplanted on May 15, 2008 (week 21), see Fig 1 for the layout and details of the greenhouse. Routine plant management was carried out, such as automatic watering with a nutrient solution and flower harvesting. To avoid influencing the thrips population with this agronomic practice, cut flowers were left close to the cutting site and in contact with the origin plant for at least two days. Pesticide applications were strictly avoided and, in order to initiate the experiment at very low or null densities of arthropods in the crops, we adopted preventive arthropod control measures and mass trapping by placing yellow sticky bands (0.3 x 6 m) among the plant rows. Traps were removed the day before the first artificial pest release.

A colony of *F*. *occidentalis* was initiated starting from individuals collected on protected rose crops and was maintained on caged rose plants in climatic chambers (24±1°C; 65% RH). The rose crop was infested homogeneously by *F*. *occidentalis* by releasing recently moulted thrips adults in the whole experimental crop. The artificial and homogenous infestation was performed through five weekly releases (from week 33 to 37) of 50 adults each (10 thrips per row) into the greenhouse compartment. Thrips were handled through a fine paint brush and gently released on young leaves.

Monitoring of thrips population dynamics was performed weekly on rose flowers using a standardized technique, the knock-down technique, which consists of tapping the rose flowers onto a white sheet of paper and counting larvae and adults [13]. Each week, 25 roses, located in the same positions as the temperature and humidity sensors, were sampled in the greenhouse using this non-destructive technique and the captured thrips were immediately released on the sampled plants. We recall that for marketable production only one rose was left per plant and that the greenhouse includes two double and one single row of plants. For double rows, the observations are the average of the two plants located around the climatic sensor sites. To sum up, there are 25 (= 5 x 2+ 5x2 + 5) observed roses corresponding to only15 (= 5+5+5) climatic sensor sites.

### Statistical models

To investigate the relationship of the infestation levels of thrips on the climate conditions and to determine their preferences regarding temperature and humidity in the actual greenhouse conditions, we specifically developed two statistical models relating climatic variates to the larva and adult thrips locations inside the greenhouse.

For each sampled monitoring site i, i = 1, …,15, and observation time t_j_ (in weeks) t_j_ = j; j = 1,…, 5., let ${\text{H}}_{\text{i},{\text{t}}_{\text{j}}}$ denotes the weekly mean of humidity, ${\text{T}}_{\text{i},{\text{t}}_{\text{j}}}$ the weekly mean of temperature, ${\text{N}}_{\text{i},{\text{t}}_{\text{j}}}^{\text{L}}$ the number of thrips larvae and ${\text{N}}_{\text{i},{\text{t}}_{\text{j}}}^{\text{A}}$ the number of thrips adults. First, we assumed that thrips populations in the different sites evolved independently (without specified spatial interactions) from each other. This did not exclude that the thrips individuals behaved similarly when moving from or settling in sites (i.e. mean behaviour but without individual interactions). Next, regarding the thrips dynamics, we sensibly assumed that it was a Markovian process in time where the present state randomly depended only on previous local climatic conditions and previous thrips population sizes. Moreover, in modelling the thrips dynamics we hypothesised the existence of optimal values (or ranges) for both temperature and humidity that favour the presence or development of thrips.

Our first model takes into consideration that the larvae resulted from egg laying events by adults that, in average, are known to occur 5.3 days before the L1 stage and 8.3 days before the L2 stage at 27°C [1]. Therefore, we considered that the density of *F*. *occidentalis* larvae at a given time *t* depends on temperature and humidity conditions where the adults were ovipositing (and feeding) at *t*-7 days. Climatic conditions at time *t*-7 has likely an influence on the egg laying activity of adults and subsequent location of the larvae at the time *t* (i.e., the propensity to successful develop from an egg), because larvae do not have wings and thus cannot move far.

In the same way, we took into consideration that the adults at time *t* were the result of egg laying events which took place 14 days earlier. We retained the climatic conditions (temperature and humidity) at *t*-14 because the required time to complete development of *F*. *occidentalis* at 25°C from oviposition to adult emergence is 14.3 days [25].

Besides, as thrips were artificially inoculated with merely two individuals, the adult and larva population sizes become noticeable only from the fourth week, and as a temporal Markovian assumption of one and two week lags was used, we only dealt with the last three observation dates regarding the first two as initial conditions and explaining covariates. Here are now the two statistical models, denoted *M* ^*L*^ (L for larvae) and *M* ^*A*^ (A for adults), devoted to the inference of some issues concerning how and to what extent larva and adult trips move and behave according to climate conditions.

#### Modelling of how larva counts statistically depend on adults and favourable climatic conditions of the week earlier?

*Model M* ^*L*^: For larva population dynamics, we assumed that at time *t*_*j*_, conditionally to previous adult population size, i.e. ${\text{N}}_{\text{i},{\text{t}}_{\text{j}}-1}^{\text{A}}$ and climatic conditions ${\text{H}}_{\text{i},{\text{t}}_{\text{j}}-1}$ and ${\text{T}}_{\text{i},{\text{t}}_{\text{j}}-1}$, the larva population size $N_{i,t_{j}}^{L}$ is Poisson distributed with mean

$$\lambda_{\text{i},{\text{t}}_{\text{j}}}^{\text{L}}\;=\;({\text{e}}^{\alpha_{0}^{\text{L}}}\;+\;{\text{e}}^{\alpha_{{}_{\text{A}}}^{\text{L}}}\;{\text{N}}_{\text{i},{\text{t}}_{\text{j}}-1}^{\text{A}})(1\;+\;{\text{e}}^{\left\{-\alpha_{\text{T}}^{\text{L}}\;{\left({\text{T}}_{\text{i},{\text{t}}_{\text{j}}-1}-{\text{m}}_{\text{T}}^{\text{L}}\right)}^{2}-\alpha_{\text{H}}^{\text{L}}\;{\left({\text{H}}_{\text{i},{\text{t}}_{\text{j}}-1}-{\text{m}}_{\text{H}}^{\text{L}}\right)}^{2}\right\}})$$ (1)

The parameter $\alpha_{0}^{L}$ or rather ${\text{e}}^{{}_{\alpha_{\text{A}}^{\text{L}}}}$, stands for a completely random contribution, (denoted hereafter CRC) in thrips larvae to account for potential immigration, larvae and adults previously present but not observed, etc. …, The component ${\text{e}}^{\alpha_{\text{A}}^{\text{L}}}{\text{N}}_{\text{i},{\text{t}}_{\text{j}}-1}^{\text{A}}$ says that an adult observed the previous week is expected to engender ${\text{e}}^{\alpha_{\text{A}}^{\text{L}}}$ larvae during the next week. The last term refers, if ever, to the climatic component. It says that the expected number of larvae increases under optimal/favourable climatic conditions (optimal temperature value ${\text{m}}_{\text{T}}^{\text{L}}$ and optimal humidity value ${\text{m}}_{\text{H}}^{\text{L}}$). It also says that temperature and humidity act independently on thrips preference and that these contributions decrease with Gaussian rates $\alpha_{\text{T}}^{\text{L}}$ and $\alpha_{\text{H}}^{\text{L}}$ as temperature and humidity departed from these optimal values. In this statistical model, the vector of the unknown parameters $\theta^{\text{L}}=(\alpha_{0}^{\text{L}},\alpha_{\text{A}}^{\text{L}},\alpha_{\text{H}}^{\text{L}},{\text{m}}_{\text{H}}^{\text{L}},\alpha_{\text{T}}^{\text{L}},{\text{m}}_{\text{T}}^{\text{L}})$ has length 6.

#### Modelling of how adult counts statistically depend on larvae present a week before and on climatic conditions present two weeks earlier?

*Model M* ^*A*^ is similar to Model *M* ^*L*^. We assumed in this case that adult population depends on larvae population ${\text{N}}_{\text{i},{\text{t}}_{\text{j}}-1}^{\text{L}}$ of the previous week but, contrarily to the previous model, it depends on the attractiveness of climatic conditions present two weeks before

${\text{H}}_{\text{i},{\text{t}}_{\text{j}-2}}$ and ${\text{T}}_{\text{i},{\text{t}}_{\text{j}}-2}$. So, the Poisson distributions of counts ${\text{N}}_{\text{i},{\text{t}}_{\text{j}}}^{\text{A}}$ of adult thrips observed at time t_j_ has the following conditional expectation:

$$\lambda_{\text{i},{\text{t}}_{\text{j}}}^{\text{A}}\;=\;\left({\text{e}}^{\alpha_{0}^{\text{A}}}\;+\;{\text{e}}^{\alpha_{\text{L}}^{\text{A}}}\;{\text{N}}_{\text{i},{\text{t}}_{\text{j}}-1}^{\text{L}}\right)(1\;+\;{\text{e}}^{-\alpha_{\text{T}}^{\text{A}}\;{\left({\text{T}}_{\text{i},{\text{t}}_{\text{j}-2}}-{\text{m}}_{\text{T}}^{\text{A}}\right)}^{2}-\alpha_{\text{H}}^{\text{A}}\;{\left({\text{H}}_{\text{i},{\text{t}}_{\text{j}-2}}-{\text{m}}_{\text{H}}^{\text{A}}\right)}^{2}})$$ (2)

Similarly, we can recover in this formula 3 components: the completely random contribution, the larva contribution and the climatic component. The corresponding parameters have the same interpretation as in the first model but refer to adult thrips. Here, the vector of parameters is denoted $\theta^{\text{A}}=(\alpha_{0}^{\text{A}},\alpha_{\text{L}}^{\text{A}},\alpha_{\text{H}}^{\text{A}},{\text{m}}_{\text{H}}^{\text{A}},\alpha_{\text{T}}^{\text{A}},{\text{m}}_{\text{T}}^{\text{A}})$ and has the same length 6.

Actually, we tested other models of dynamics which revealed to be statistically “unstable” due to the relatively low size of the experimental data set (in time and in space). The two models proposed here for larva and adult populations of thrips revealed numerically stable proving that enough informative data was presently provided in order to test some relevant hypotheses.

#### Parameter Estimation and hypothesis testing

The assumption that local thrips populations were independent from plant to plant and behaved according to a Markovian dynamics with the previous form of conditional Poisson distributions makes it possible to express the likelihood of the observed data. For simplicity, we only write down the log-likelihood of Model *M* ^*L*^ (otherwise replace *L* by *A* for model *M* ^*A*^):

$$\text{ln}(\theta^{\text{L}})=\sum_{\text{i}=1}^{15}\sum_{\text{j}=3}^{5}(-\lambda_{\text{i},{\text{t}}_{\text{j}}}^{\text{L}}+{\text{N}}_{\text{i},{\text{t}}_{\text{j}}}^{\text{L}}\;\text{log}(\lambda_{\text{i},{\text{t}}_{\text{j}}}^{\text{L}})$$ (3)

First, for the estimation issue, the maximum likelihood estimators ${\hat{\theta }}^{\text{L}}$ and ${\hat{\theta }}^{\text{A}}$ in Table 1 were computed using library “bbmle” of R Software (R Development Core Team 2005). Note that the same subroutine also yields the approximate standard deviations of the parameters.

**Table 1:** Parameter estimates in full models for larva and adult presence in relation to climatic and other covariates.

**Full Model M** ^**L**^ **(Larvae)** $-2\;\text{ln}({\hat{\theta }}^{\text{L}})=\;-298.8826$			**Full Model M** ^**A**^ **(Adults)** $-2\;\text{ln}({\hat{\theta }}^{\text{A}})=\;-187.908$		
*Parameter*	*Estimate*	*Std*. *Error*	*Parameter*	*Estimate*	*Std*. *Error*
$\alpha_{0}^{\text{L}}$	-0.155	0.210	$\alpha_{0}^{\text{A}}$	1.264	0.087
$\alpha_{\text{A}}^{\text{L}}$	0.039	0.114	$\alpha_{\text{L}}^{\text{A}}$	-0.665	0.363
${\text{m}}_{\text{T}}^{\text{L}}$	22.004	0.119	${\text{m}}_{\text{T}}^{\text{A}}$	27.010	0.104
$\alpha_{\text{T}}^{\text{L}}$	1.893	4.748	$\alpha_{\text{T}}^{\text{A}}$	5.298	9.44e-06
${\text{m}}_{\text{H}}^{\text{L}}$	86.380	0.745	${\text{m}}_{\text{H}}^{\text{A}}$	62.790	0.215
$\alpha_{\text{H}}^{\text{L}}$	0.045	0.023	$\alpha_{\text{H}}^{\text{A}}$	1.24	5.78e-05

Next, concerning hypothesis testing, if we let the sign * stand for L (larvae) or A (adults), we tested several specific hypotheses, say “$H_{0X}\;:\theta^{*}\in \Theta_{0\text{X}}^{*}$” (corresponding to a sub-model $M_{0X}^{*}$) vs. the alternative $\theta^{*}\notin \Theta_{0\text{X}}^{*}$(within the global model *M* ^*^), using the classical asymptotic log likelihood ratio test statistic $\Delta_{0\text{X}}^{*}=-2(\text{ln}({\hat{\theta }}_{{\text{M}}_{0\text{X}}^{*}})-\text{ln}({\hat{\theta }}_{{\text{M}}^{*}}))$. Statistical theory asserts that under the assumptions defining sub-model $M_{0X}^{*}$, the statistic $\Delta_{0X}^{*}$ is asymptotically χ^2^(k) distributed, k being the difference of the dimensions of parameter spaces under *M* ^*^ and $M_{0X}^{*}$ [34]. The significance of all our tests referred to the level of 1%. Actually, we gave the values of $\Delta_{0X}^{*}$ and the quantiles ${\text{P}}_{\chi^{2}(\text{k})}^{-1}(0.99)$ of the significance levels to better see the departure from the corresponding p-values.

In this paper, we tested for both models three types of hypotheses. (1) We tested the non-effectiveness of the completely random contribution (CSC), that is $M_{0R}^{*}\;{}^{"}\alpha_{0}^{*}\approx -\infty^{"}$or rather more practically $\alpha_{0}^{*}=-12$ so that $e^{\alpha_{0}^{*}}\leq 10^{-5}$. (2) We also tested the non-effectiveness of larva (resp. adult) contribution to adult presence (resp. larva presence) that is $M_{0L}^{A}:\;{}^{"}\;\alpha_{0L}^{A}\approx -\infty^{"}$, or rather $\alpha_{0L}^{A}=-12$ so that $e^{\alpha_{0L}^{A}}\leq 10^{-5}$ (resp. $M_{0A}^{L}:\;{}^{"}\;\alpha_{0A}^{L}\approx -\infty^{"}$, or rather $\alpha_{0A}^{L}=-12$ so that $e^{\alpha_{0A}^{L}}\leq 10^{-5}$). The corresponding test statistic $\Delta_{0X}^{*}$ is expected to behave as a *χ* ^2^(1) distribution under the respective sub-models (1) and (2).

Finally, we tested (3) the global non-effectiveness of the climate component, via the sub-model $M_{0C}^{*}$ assuming the hypothesis: “${\text{e}}^{-\alpha_{\text{T}}^{*}\;{\left({\text{T}}_{\text{i},{\text{t}}_{\text{j}}}-{\text{m}}_{\text{T}}^{*}\right)}^{2}-\alpha_{\text{H}}^{*}\;{\left({\text{H}}_{\text{i},{\text{t}}_{\text{j}}}-{\text{m}}_{\text{H}}^{*}\right)}^{2}}=0$”. From a practical point of view, this is performed by assuming: $\alpha_{T}^{*}=\alpha_{H}^{*}=50,\;m_{T}^{*}=10,\;m_{H}^{*}=50$so that ${\text{e}}^{-\alpha_{\text{T}}^{*}\;{\left({\text{T}}_{\text{i},{\text{t}}_{\text{j}}}-{\text{m}}_{\text{T}}^{*}\right)}^{2}-\alpha_{\text{H}}^{*}\;{\left({\text{H}}_{\text{i},{\text{t}}_{\text{j}}}-{\text{m}}_{\text{H}}^{*}\right)}^{2}}\;\approx 0$ whatever are the values taken by $T_{i,t_{j}}\;,\;H_{i,t_{j}}$ and in this case, $\Delta_{0C}^{*}$should behave as a *χ* ^2^(4) under hypotheses defining $M_{0C}^{*}$.

## Results

### Experimental results

**Fig 2:**
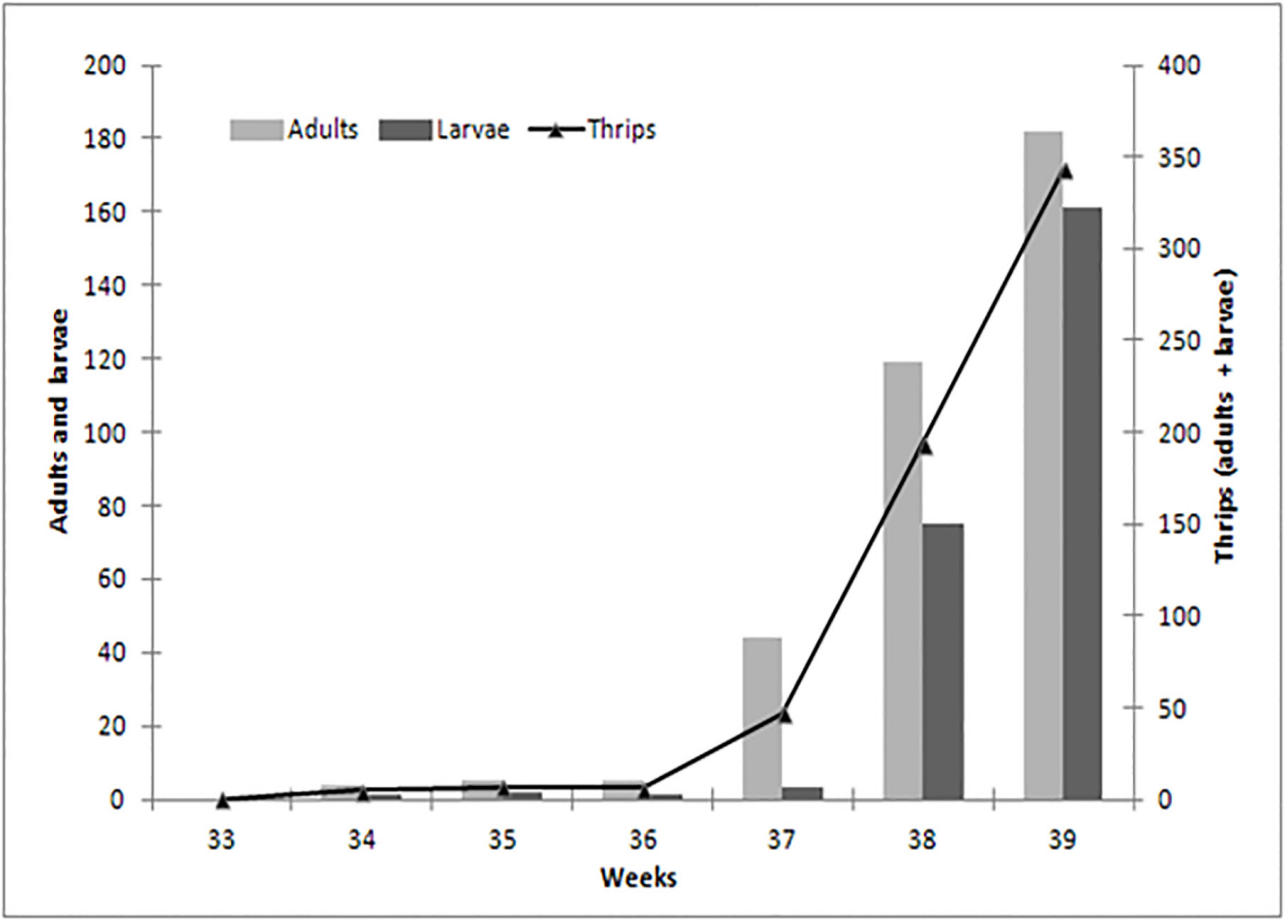
Time evolution of larvae and adults population of thrips in the experimental crop.

The thrips populations began to increase slowly after the start of the experiment in calendar week 33. From week 37 on, there was a sudden and persistent increase of the number of thrips throughout the experiment until its end in week 39, then the number of thrips larvae gradually increased from week 37 to 39 (respectively 3, 75 and 161 larvae) together with the number of adults: 44, 119 and 182 adults (Fig 2). During weeks 38 and 39, the number of adult thrips and larvae increased 4 and 7 fold, respectively. At the end of week 39 the mean number of thrips per flower was equal to 13.7 and the experiment was ended, because this level was higher than the admitted economic threshold [13]. No thrips natural enemies or other pests were found on the experimental crop during the whole experiment.

**Fig 3:**
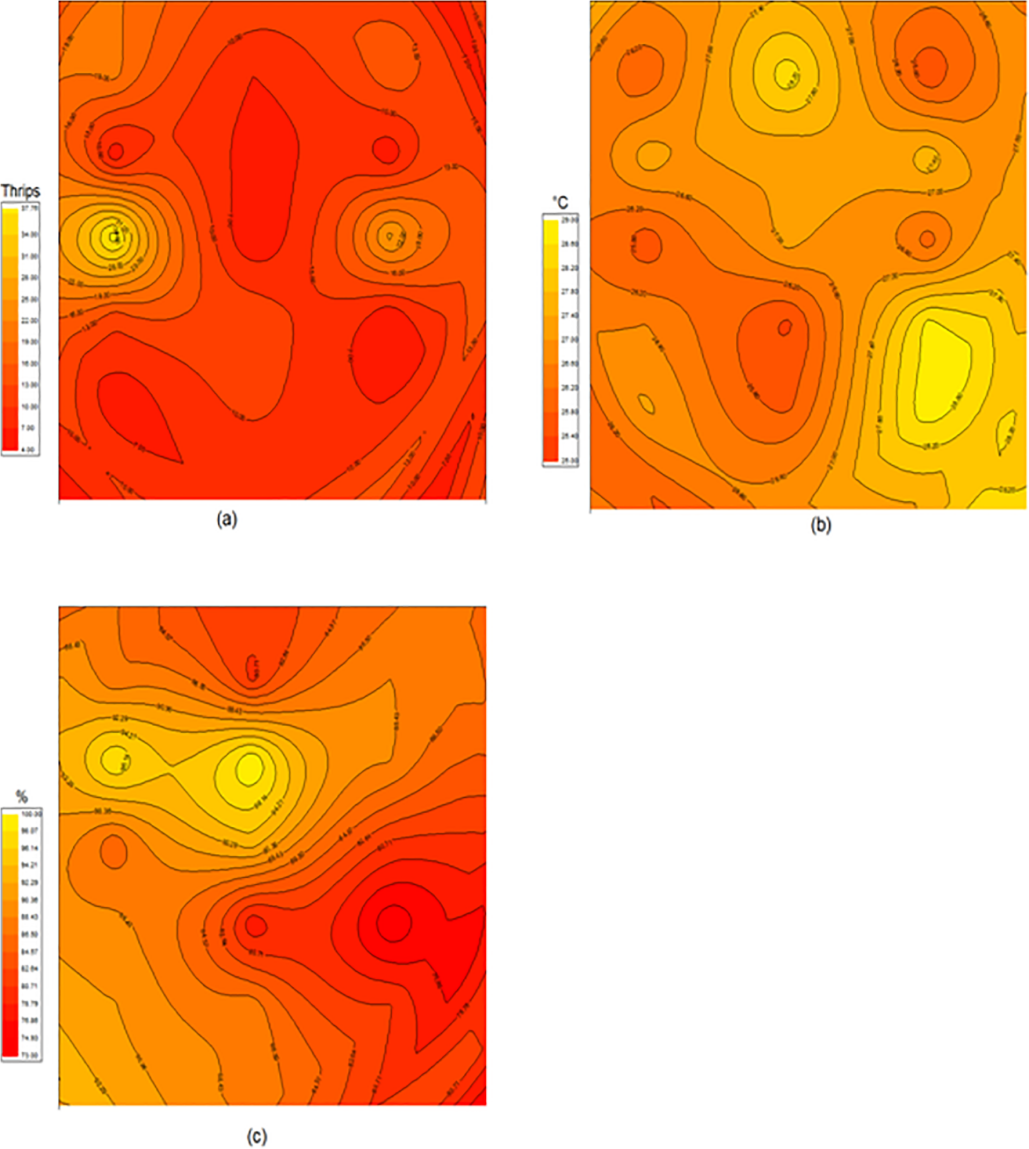
Contour maps of the (a) thrips population size (b) weekly mean diurnal temperature and (c) weekly mean air humidity within the experimental crop.

The thrips population was not distributed homogeneously within the crop cover (Fig 3A). It was higher in the south and in the north and lower in the central part of the compartment. The climatic conditions (diurnal temperature and humidity respectively) inside this greenhouse compartment measured in a horizontal plane located at 1.3 m above ground were unevenly distributed (Fig 3B and 3C). The diurnal temperature varied between 25 and 29°C (Fig 3A). Air humidity was also heterogeneous inside the greenhouse with the highest values observed in the central part. The lowest humidity occurred along the north-western part of the greenhouse.

### Mapping larva and adult densities with respect to climatic conditions

**Fig 4:**
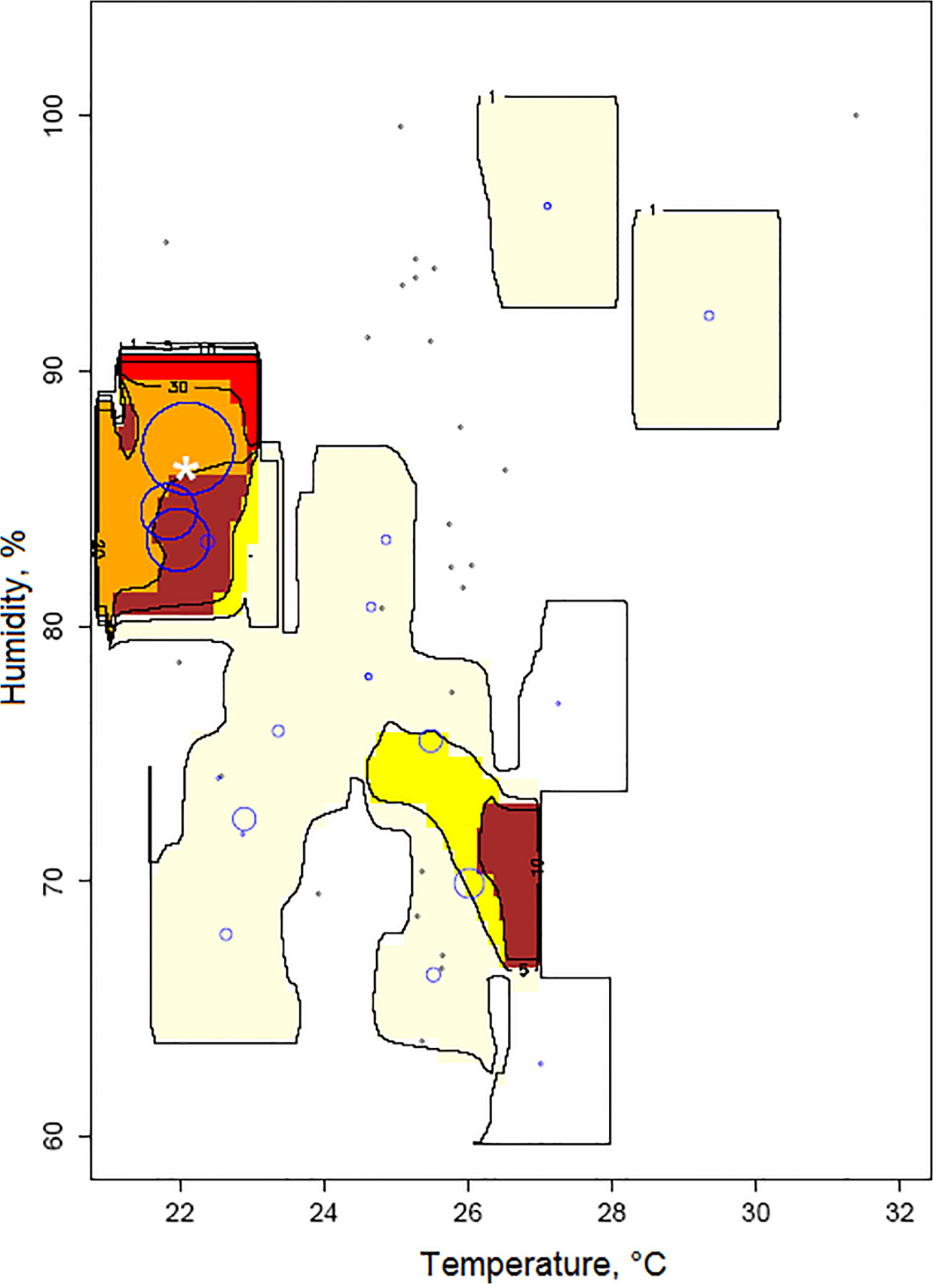
Within-crop distribution of thrips larvae in relation to the temperature and humidity. The size of circles corresponds to the number of larvae observed over the last three weeks. The * sign corresponds to estimated preferred climatic conditions.

**Fig 5:**
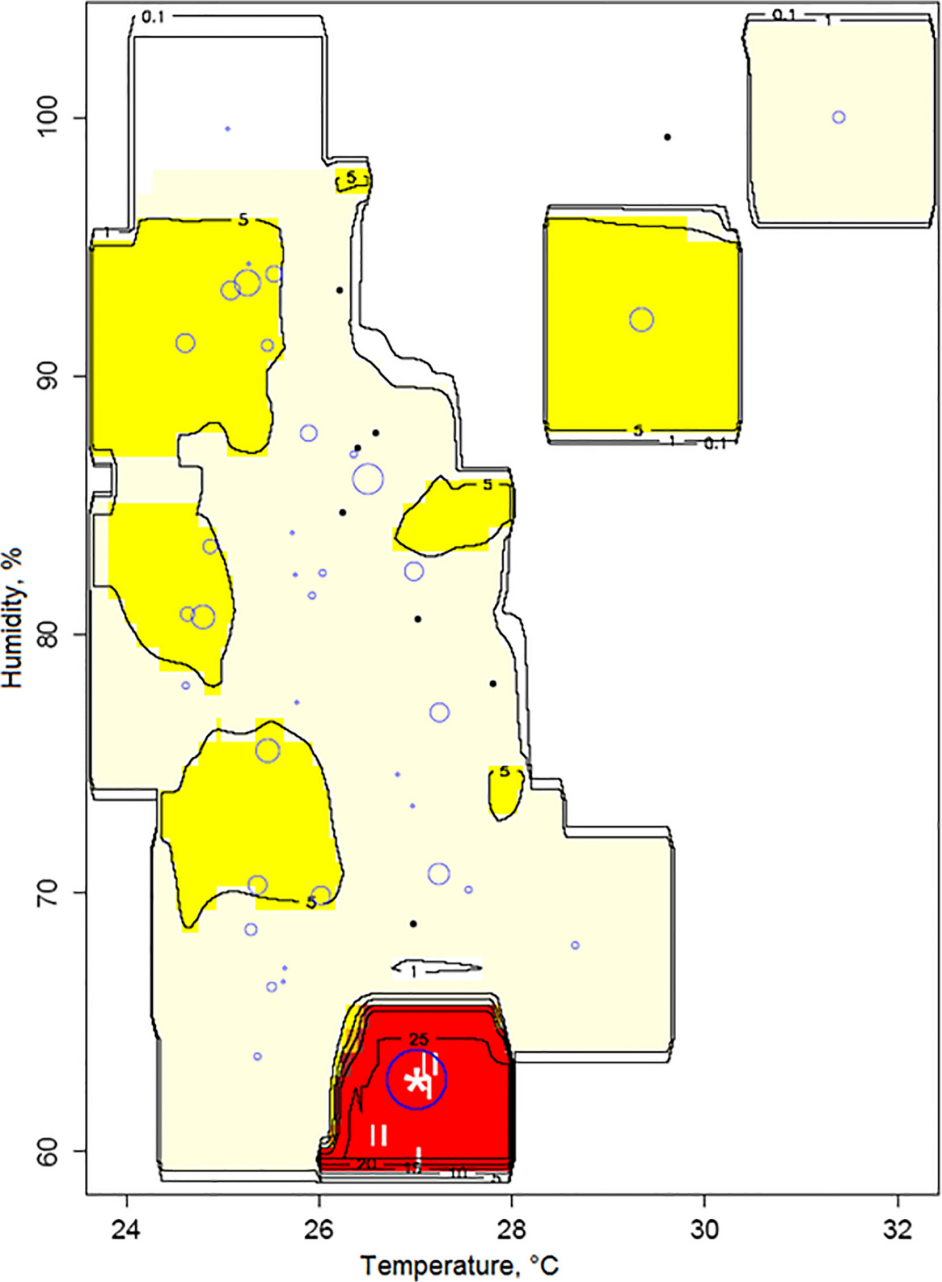
Within-crop distribution of thrips adults in relation to the temperature and humidity. The size of circles corresponds to the number of adults observed over the last three weeks. The * sign corresponds to estimated preferred climatic conditions.

The density map of thrips larvae with respect to air temperature and humidity (Fig 4) showed that the number of thrips larvae was high in spatial areas whose temperature and humidity are around 22°C and 86%, and decreased outside these zones. Similarly, when comparing the distribution pattern of thrips adults with respect to temperature and humidity (Fig 5), the highest population density of thrips adults was recorded at 27°C for temperature and 63% for humidity

### Statistical results

Recall first that, the significance of all our tests referred to a level of 1% to which corresponds a quantile ${\text{P}}_{\chi^{2}(\text{k})}^{-1}(0.99)$. The statistical results concerning hypothesis testing (or equivalently of submodels) are summed up in Tables 2 and 3. So, for both models, the effectiveness of a complete random contribution is highly significant. More specifically, for larva presence, we obtained:

$\Delta_{0\text{R}}^{\text{L}}=-2(\text{ln}({\hat{\theta }}_{0\text{R}}^{\text{L}})-\text{ln}({\hat{\theta }}^{\text{L}}))=369.46\gg 6.64={\text{P}}_{\chi^{2}(1)}^{-1}(0.99),\;({\text{p}}_{\text{value}}\approx 0)$ and for adult presence we obtained an even greater significance $\Delta_{0\text{R}}^{\text{A}}=-2(\text{ln}({\hat{\theta }}_{0\text{R}}^{\text{A}})-\text{ln}({\hat{\theta }}^{\text{A}}))=2023.5\gg 6.64={\text{P}}_{\chi^{2}(1)}^{-1}(0.99),\;({\text{p}}_{\text{value}}\approx 0)$.

Next, concerning the contribution of the adults present a week before on the larva presence of the current weak revealed also highly significant: $\Delta_{0\text{A}}^{\text{L}}=-2(\text{ln}({\hat{\theta }}_{0\text{A}}^{\text{L}})-\text{ln}({\hat{\theta }}^{\text{L}}))=126.7\gg 6.64={\text{P}}_{\chi^{2}(1)}^{-1}(0.99)(\text{i}.\text{e}.\;{\text{p}}_{\text{value}}\approx 0)$. Similarly, the contribution of larvae present a week before on adult presence of the current weak, even if lesser, was also highly significant: $\Delta_{0\text{L}}^{\text{A}}=-2(\text{ln}({\hat{\theta }}_{0\text{L}}^{\text{A}})-\text{ln}({\hat{\theta }}^{\text{A}}))=34\gg 6.64={\text{P}}_{\chi^{2}(1)}^{-1}(0.99),\;(\text{i}.\text{e}.\;{\text{p}}_{\text{value}}\approx 0)$

**Table 2:** Larva presence: Hypothesis testing on covariate effect.

Sub Models M^L^_0X_	$-2\;\text{ln}({\hat{\theta }}_{0\text{X}}^{\text{L}})$	$-2(\text{ln}({\hat{\theta }}_{0\text{X}}^{\text{L}})-\text{ln}({\hat{\theta }}^{\text{L}}))∼\;\chi^{2}\left(\;\text{k}\right)$	P-value
$H^{L}{}_{0R}:\alpha_{0}^{\text{L}}=-\infty$ “no CR contribution”	70.58	369.46 ~ χ^2^ (1)	0
$H^{L}{}_{0A}:\alpha_{\text{A}}^{\text{L}}=-\infty$ “no adult contribution”	-172.19	126.69 ~ χ^2^ (1)	0
***H*** ^***L***^_***0C***_: “no climatic effect”	**-238.566**	60.3 ~ χ^2^ (4)	0

**Table 3:** Adult presence: hypothesis testing on covariate effect.

Sub Models M^A^_0X_	$-2\;\text{ln}({\hat{\theta }}_{0\text{X}}^{\text{A}})$	$-2(\text{ln}({\hat{\theta }}_{0\text{X}}^{\text{A}})-\text{ln}({\hat{\theta }}^{\text{A}}))∼\;\chi^{2}\left(\;\text{k}\right)$	P_value
$H^{A}{}_{0R}:\alpha_{0}^{\text{A}}=-\infty$ “no CR contribution”	2015.58	2023.5 ~ χ^2^ (1)	0
$H^{A}{}_{0A}:\alpha_{\text{L}}^{\text{A}}=-\infty$ «no larva contribution »	-153.06	34 ~ χ^2^ (1)	0
***H*** ^***A***^_***0C***_ **: “**no climate effect”	-161.45	26.46 ~ χ^2^ (4)	10^–5^

Finally, for the effectiveness of climate effects, the tests revealed also very significant. When testing the contribution in model *M* ^*L*^ we got $\Delta_{0\text{C}}^{\text{L}}=-2(\text{ln}({\hat{\theta }}_{0\text{C}}^{\text{L}})-\text{ln}({\hat{\theta }}^{\text{L}}))=60.32∼\chi (4),\;(\text{i}.\text{e}.\;{\text{p}}_{\text{value}}\approx 0)$ and in model *M* ^*A*^ $\Delta_{0\text{C}}^{\text{A}}=-2(\text{ln}({\hat{\theta }}_{0\text{C}}^{\text{A}})-\text{ln}({\hat{\theta }}^{\text{A}}))=26.45∼\chi (4),\;(\text{i}.\text{e}.\;{\text{p}}_{\text{value}}\leq 10^{-4})$.

Since the full models *M* ^*L*^ and *M* ^*A*^ proved to be statistically justified regarding the effectiveness of the three distinct components, we turn now to the quantification of these contributions, that is, to parameter estimation issues (Table 1). So, for larva presence (model *M* ^*L*^), the completely random contribution on larva presence was about exp(-0.155) = 0.86 larvae per site and per week. Concerning the contribution of one single adult previously present, we obtained exp(0.039) = 1.04 larvae per adult. As for the optimal conditions, we got 22.00°C for temperature and 86.38% for humidity. With these most favourable climatic conditions, the contributions were twice. The values of the Gaussian decreasing rates in *M* ^*L*^ were estimated to 1.89 for temperature and to 0.045 for humidity. Note that even if we took account of the difference between temperature and humidity ranges, the Gaussian decrease rate was more abrupt for temperature than for humidity.

For adult presence (model *M* ^*A*^), there were in average a completely random contribution of exp(1.26) = 3.54 adults per site per week and that one previously observed larva yielded exp(-0.665) = 0.51 adults in mean for the current week. As for the optimal climatic conditions for adults, these were 27.01°C for temperature and 62.79% for humidity. Under these optimal conditions, the number of adults doubled. Similarly, note the higher value of the Gaussian rate for temperature 5.298 compared to the Gaussian rate for humidity 1.24. Note also the high values of these Gaussian rates in adult model when compared to those of larva model. The higher the values of the Gaussian decreasing rates, the smaller are the favourable climatic zones.

## Discussion

Fine scale climate measurements and thrips distributions within a greenhouse rose crop were monitored to investigate the dependence of thrips infestation on climate conditions. For that purpose, a specific statistical model relating to space-time locations of thrips inside greenhouse was used to define the favourable climate conditions for thrips adults and larvae. On the one hand, data showed that air temperature and air humidity were very heterogeneously distributed inside the greenhouse. This heterogeneity was likely due to the difference of the transpiration activity of plants and air circulation inside the greenhouse [31]. On the other hand, our results highlighted that diurnal temperature and humidity exert a direct effect on the distribution of thrips (*F*. *occidentalis*) within the greenhouse crop. More precisely, we found that the non-homogeneous distribution of thrips infestation inside the greenhouse was partly due to the research of favourable climatic zones (Tables 2 & 3).

**Fig 6:**
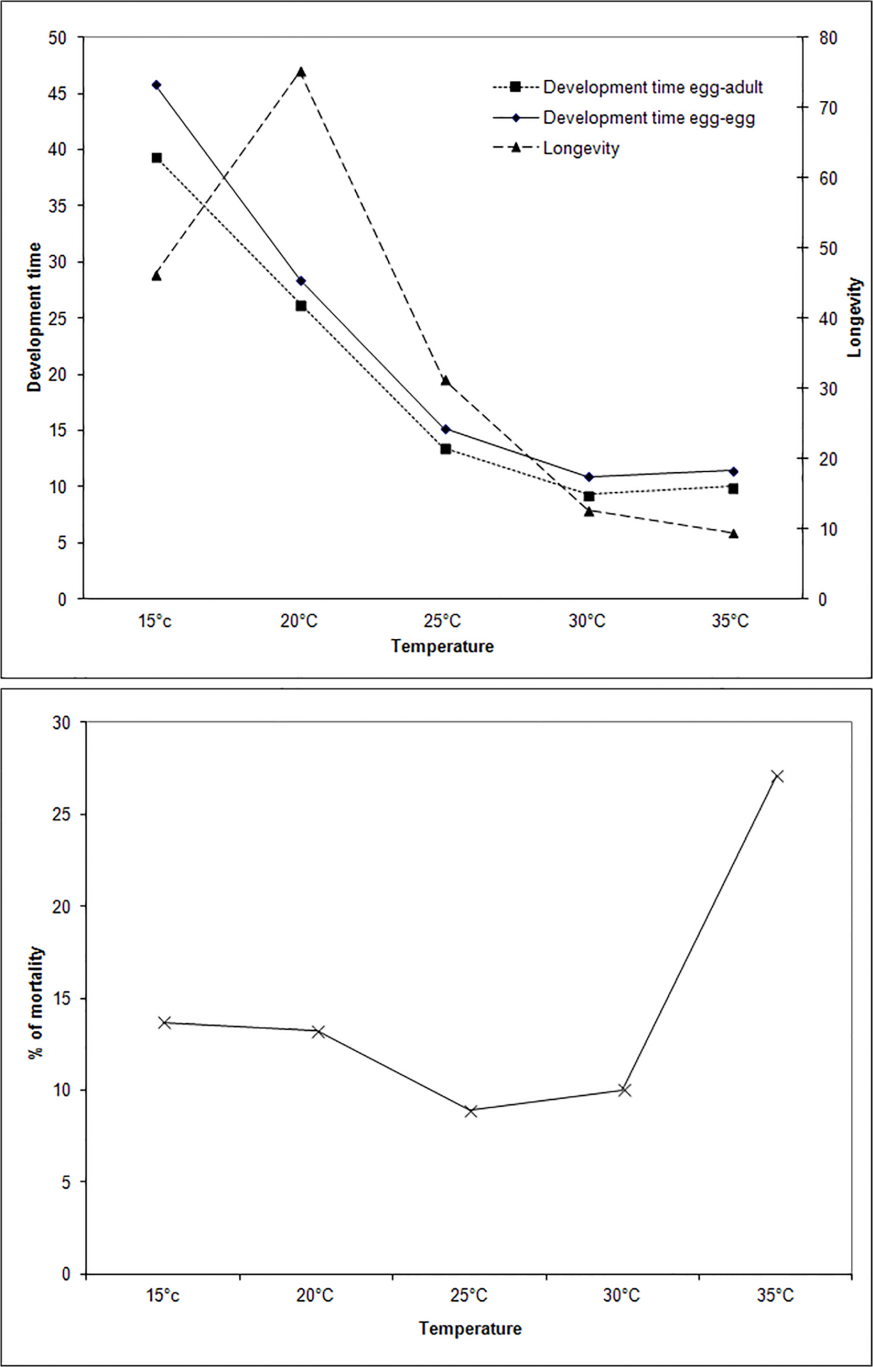
Dependence of development time, longevity (a) and mortality (b) of *F*. *occidentalis* on temperature feeding on chrysanthemum (modified from Robb, 1989).

The dependence of *F*. *occidentalis* from temperature regimes has been demonstrated under well-controlled laboratory conditions by Robb [35], Robb & Parella [28] and Brodsgaard [36]. They showed how thrips gain an optimum development between 25°C and 27°C and that the female laying inflates from 133.6 eggs at 25°C to 228.6 eggs at 27.2°C. The increase of the *F*. *occidentalis* population density can also be due to the conjunction of a greater longevity at 27.2°C almost three times more than at 30°C according to Robb and Parella [28], a shorter development period (see Fig 6A) and a lower mortality rate (see Fig 6B) [35]. These studies are in line with our results relating to the temperature preference of adult thrips around 27°C (Fig 5). Within this favorable temperature, the rapid development of *F*. *occidentalis* and the high fertility of females, associated with a low mortality rate, had probably a significant effect on the rate of the pest population increase. In addition, our results yielded the mean favourable temperature for larvae.

Concerning the influence of humidity, Steiner et al. [37] and Shipp & Gillespie [38] showed that all stages of *F*. *occidentalis* are sensitive to humidity. The humidity range between 65% and 85% was unfavourable to the larvae and more favourable to the pupae and adults [37]. In this context, our statistical results were more precise in the sense that they pinpointed the optimal humidity values of 86% for larvae and 63% for the adults.

The higher concentration areas of thrips (adults or larvae) found in the greenhouse (Fig 3), may be due to the concomitance of two phenomena acting at two distinct scales: (i) locally, the higher population reproduction rate induced by optimal environmental conditions, and (ii) at a larger scale, the thrips displacements and in particular to areas with more favourable conditions. However, although the dispersal ability of *F*. *occidentalis* females has been estimated to be 0.18 to 0.29 m per day [39] corresponding to a maximum of 2 m per week in our study time scale, further specific tests on the *F*. *occidentalis* dispersal capacity on rose crop should be carried out to support this hypothesis. Indeed, the dispersal ability of insects can largely vary with the attributes and availability of their hosts [40, 41]. For this lack of information, these dispersal capacities were simply integrated in our models in what was defined as the completely random contributions. Although the estimated values were very different for larvae (0.86 larvae per site per week) and adult (3.54 adults per site per week), these random contributions revealed both very significant.

In addition, our results allowed the prediction of the weekly contributions of present adults (resp. larvae) to larva (resp. adult) presence for the next week. For example, in our study, with an average of 7 adults per site per week, the contribution was about 7x1.04 = 7.28 larvae. This value is very high compared to the random contribution of 0.86 larvae. Conversely, with an average of 5.3 larvae per site per week, the contribution was about 5.3x0.51 = 2.7 adults which is lower than the random contribution of 3.54 adults. These figures seemed to simply translate the action of the two mentioned scales, local climatic preferences and adult dispersal, as well as their respective importance.

Concerning the attractiveness rates toward the favorable climatic conditions, we first observed that, even if took account of the difference between the temperature and humidity ranges, the temperature contribution was more rapidly decreasing (three times) than the humidity contribution when departing from the favorable conditions in the two models. Second, we also observed that these decrease rates are very high for adults that for larvae suggesting that this can be due to the dispersal capacity of adults in their search of favorable conditions.

Besides validating, in real crop conditions, the life-table data generated in the laboratory [35], our findings could be put to good use to propose optimal greenhouse microclimate conditions combined with agricultural practices which consider both thrips limitations as well as plant health. This fully fits with the main principles of Integrated Pest Management (IPM) which combines prophylactic, biological and physical methods against bioagressors of crops, while minimizing the use of pesticides [42,43,18]. Indeed, cultural (agronomic and physical) control techniques developed from crop management or mechanical practices can be adapted to disadvantage pest population growth while having limited adverse effects on crop productivity. Our findings also suggest that once the climatic preferences of thrips are identified, the infestation can be effectively minimised by releasing natural enemies in small areas surrounding the *risk zones*, saving time for the infestation monitoring and amount of natural enemies to be released. In contrast, before giving overall conclusions on optimal microclimatic conditions to achieve within protected rose cultivations, further specific tests should be carried out to assess the effects on the thrips dynamics of other physical factors, e.g. the luminosity, airflows, …. Similar studies should be carried out focusing on other rose pests and diseases as well as on their interactions [44] according to various microclimatic regimes.

## Conclusion

The favourable climatic conditions for thrips (*F*. *occidentalis*) adults and larvae were assessed from the comparison of the distribution of temperature, humidity and thrips within the greenhouse. Our results highlighted that diurnal temperature and humidity exert a direct effect on the distribution of thrips (*F*. *occidentalis*) within the greenhouse space.

These findings could be used to define a climate control strategies to fight against this insect by modifying temperature and humidity inside the greenhouse to make it unfavourable for this pest. It may also be used to identify the risk areas, and thus saving time and money for growers, by releasing natural enemies in small areas surrounding these risk zones. To be complete on the topic, photoperiod effect must be also investigated and the Computational Fluid Dynamics (CFD) simulation tool could be will be used to draw a risk maps inside greenhouse according to the climatic parameters. CFD could also help to define how to change the local climate at the crop level to turn them unfavourable for pests.
